# Obituary

**Published:** 1856-04

**Authors:** 


*Obituary.*—Died, at his residence, at Fond du Lac, Wisconsin, on the 31st of January last, Dr. Rufus W. Root, aged 28 years.

Dr. Root was a graduate of the first class of the University of Buffalo, and was a brother of Dr. John Root, of this city, known to our readers as the late Health Physician.

Dr. R., owing to ill-health, practised his profession for a short time only. His disease was tuberculosis, coming on subsequent to a chronic pleurisy of several years standing.

